# Exploratory Evaluation of Learning Behaviors Using a ChatGPT-Based Question-Generating Bot Among Occupational Therapy Students Preparing for the National Licensure Exam: A One-Month Pre-post Study With Cluster Analysis

**DOI:** 10.7759/cureus.94447

**Published:** 2025-10-13

**Authors:** Kengo Kohiyama, Shogo Sawamura, Takashi Nagai, Takahiro Takenaka, Tatsuya Sera, Tadatoshi Inoue

**Affiliations:** 1 Department of Rehabilitation, Heisei College of Health Sciences, Gifu, JPN

**Keywords:** chatgpt, large language models, learning behaviors, licensure examination, occupational therapy

## Abstract

Introduction: This exploratory study examined occupational therapy (OT) students’ learning behaviors using a custom-built ChatGPT version 4.0 (OpenAI, Inc., San Francisco, United States) based question-generating bot. Particularly, this study examined changes in mock examination scores before and after one month of use, correlations between usage logs and score improvements, and learning behavior clusters to identify distinct engagement patterns.

Methods: This study comprised 29 OT students, using a one-group pre-post design. The intervention comprised one month of access to a ChatGPT-based bot, developed to generate multiple-choice questions in anatomy, kinesiology, and physiology. Participants were instructed to use the bot as part of their self-study and submit weekly reports via Google Forms (Google LLC, Mountain View, California, United States), documenting the number of questions generated, the number of sessions, and total usage time. Performance was assessed using two 50-item mock exams administered before (Exam I) and after (Exam II) the intervention. Paired t-tests were used to compare the exam scores. Correlations were calculated between the score gains (Exams II-I) and the learning logs. Ward’s hierarchical cluster analysis was performed to explore the distinct patterns of learning behavior.

Results: In total, 28 datasets were analyzed. Exam scores significantly improved between Exam I (17.1 ± 4.4) and Exam II (22.1 ± 7.6), with a mean gain of +5.0 ± 6.0 (t-statistic=4.40, degrees of freedom=27, p<0.001, Cohen’s d=0.83). Correlation analysis indicated weak-to-moderate associations between Exam II scores and the number of questions (r=0.39, p<0.05) and sessions (r=0.36, p<0.10), but no significant association with total usage time. Cluster analysis identified three groups: Cluster 1 (n=14) with low activity and minimal gains; Cluster 2 (n=9) with more questions but little improvement; and Cluster 3 (n=6) with moderate activity but high total usage time, achieving the greatest improvements.

Conclusions: This exploratory study examined OT students’ learning behaviors when using a ChatGPT-based question-generating bot. The findings suggest that increased total time investment may be more relevant to performance gains than the generated question quantity. Future studies should incorporate qualitative analyses of the learning processes and controlled comparisons to further validate these patterns.

## Introduction

Large language models (LLMs), such as ChatGPT (OpenAI, Inc., San Francisco, United States), have recently attracted considerable attention in health education. Regarding medicine, GPT models have been evaluated on the United States Medical Licensing Examination and the Japanese Medical Licensing Examination, achieving performance levels that suggest their potential utility as study aids [1,2]. Regarding nursing education, the introduction of LLMs contributes to the development of critical thinking through interactive simulation and the generation of clinical scenarios [3]. In addition, a previous scoping review summarized the overall use of ChatGPT in nursing, emphasizing its applications in education, practice, and research [4].

However, their application in occupational therapy (OT) is limited. Although artificial intelligence (AI)-assisted program planning has been examined, concerns persist regarding its specificity and individualization [5]. LLMs were recently evaluated for their ability to respond to occupational and physical therapy licensure examinations. For the Japanese National Physical Therapist Examination, ChatGPT-4 achieved an overall accuracy of 73.4% across five full examinations, including 80.5% for text-only items and 35.4% for items requiring the interpretation of images or tables [6]. In the 59th examination, OpenAI-o1 scored 97.0% for text-based items, whereas GPT-4o scored 56.5% for image-based items [7]. Regarding OT, GPT-4 demonstrated correct answer rates of 76%-81% on recent Japanese national examinations, which were substantially higher than ChatGPT-3.5, which remained in the mid-50% range [8]. Furthermore, LLMs have been evaluated for their ability to generate multiple-choice questions (MCQs) that resemble those used in licensure examinations [9].

Beyond examination performance, the application of AI in OT education has begun to emerge. AI-assisted intervention planning during fieldwork enhances student creativity and reduces cognitive load [5]. Experts rated neurological case vignettes generated by ChatGPT as clinically realistic and educationally useful [10]. A survey of graduate OT students reported positive perceptions concerning the usefulness of ChatGPT and its potential role in academic learning [11]. These studies suggested that LLMs may support creativity by expanding intervention options, enhancing realism through clinically plausible case vignettes, and fostering student engagement by being perceived as useful learning aids [5,10,11]. Simultaneously, concerns remain regarding the phenomenon of “hallucination,” in which LLMs generate inaccurate or misleading information. This limitation was emphasized in both medical education and clinical applications, emphasizing the need for cautious integration [12,13].

However, little is known about how OT students interact with AI tools for licensure exam preparation and how such usage behaviors influence learning outcomes. Previous studies predominantly focused on the accuracy of AI responses or students’ subjective perceptions; however, objective analyses of learning processes, such as session counts, time spent, and the number of questions generated, remain scarce. Such process-level data are crucial for providing evidence-based guidance for the effective integration of AI into exam preparation.

Therefore, this study aimed to explore the learning behaviors of OT students using a custom-built ChatGPT-based question-generating bot. Particularly, this study examined changes in mock examination scores before and after one month of use, correlations between usage logs and score improvements, and learning behavior clusters to identify distinct engagement patterns.

## Materials and methods

Study design

The study was conducted at Heisei College of Health Sciences, Gifu City, Gifu Prefecture, Japan. This study employed a single-group pretest-posttest design. The intervention lasted for one month, during which the students used a custom-built ChatGPT-based (version 4.0) question-generating bot.

Participants

The sample comprised 29 third-year OT students from a junior college in Japan. All participants provided written informed consent. One participant was excluded owing to not completing both the pre- and post-tests and failing to submit weekly usage reports, resulting in 28 datasets for analysis. 

Intervention

Bot Design and Functions

This study developed a custom-built ChatGPT-based question-generating bot. The bot was conditioned with publicly available items from the 2024 and 2025 Japanese National Examination for Occupational Therapists, which were used as references to align style and difficulty, but not for memorization or model training. The bot generated five-option MCQs limited to anatomy, kinesiology, and physiology, consistent with the licensure exam format. Students could enter up to three keywords (e.g., “shoulder,” “joint,” and “adduction”), and the bot returned one MCQ per request.

After the students submitted an answer, the bot displayed the correct option, along with a brief explanation. Typing “hint” produced a clue without disclosing the correct answer. A supplementary web search function was enabled when necessary to retrieve basic factual information, with the outputs labeled accordingly. To ensure content validity, multiple OT faculty members tested the bot before deployment and confirmed that it generally produced appropriate questions.

Student Training and Usage Instructions

All participants attended an orientation session before the intervention, in which the faculty members demonstrated the bot’s operation and explained its expected usage. Additionally, each participant received a printed manual describing the commands and reporting procedures (Appendix 1). Participants were explicitly instructed that ChatGPT outputs were not always accurate; if they encountered questionable content, they were required to verify it using textbooks or peer-reviewed sources. Furthermore, participants were reminded that no patient-identifiable information should be entered or generated.

Participants accessed the bot via a dedicated uniform resource locator (URL) using their laptops or smartphones. Moreover, they were required to submit weekly reports via Google Forms (Google LLC, Mountain View, California, United States) throughout the one-month intervention.

Outcome Measures

Learning outcomes were assessed using two 50-item mock examinations, Exam I (pre-intervention) and Exam II (post-intervention). Both examinations consisted of previous OT national exam questions limited to anatomy, kinesiology, and physiology. Three faculty members with extensive experience in licensure exam preparation selected the 50 items for each examination to ensure comparable difficulty levels. Scores were recorded as the number of correct answers.

Learning Logs

Weekly reports via Google Forms provided data on the following three indicators: total number of questions generated, total number of sessions, and total usage time (minutes).

Statistical analysis

Paired t-tests were conducted to compare Exam I and Exam II scores. Pearson’s correlation coefficients were calculated to examine the associations between exam performance (Exam II scores and gain scores, defined as Exam II - Exam I) and learning log indicators. Ward’s hierarchical cluster analysis was performed using standardized log variables (number of questions, sessions, and total time) to explore the distinct learning behavior patterns. The number of clusters was determined based on dendrogram inspection, group interpretability, and sample size (Appendix 2). Subsequently, descriptive statistics (means and standard deviations) for each cluster were calculated for exam scores (Exam I, Exam II, and gain scores) and learning log indicators. All analyses were conducted using HAD statistical software, version 18 (Shimizu, H., Kwansei Gakuin University, Japan) [14].

Ethics

This study was approved by the Ethics Committee of Heisei College of Health Sciences (approval number: R6-6) and was conducted in accordance with the Declaration of Helsinki. All participants provided written informed consent before participation.

## Results

Participant characteristics

A total of 29 third-year OT students (five males, 24 females; mean age=20.7 ± 1.0 years) participated in this study. One student did not complete the post-test, resulting in 28 complete datasets for analysis.

Descriptive statistics

During the one-month intervention, students generated an average of 31.5 ± 20.2 questions, engaged in 13.6 ± 5.7 sessions, and spent 153.1 ± 112.9 minutes using the ChatGPT-based bot. The mean score for Exam I was 17.1 ± 4.4, whereas the mean score for Exam II was 22.1 ± 7.6. The average gain (Exam II − Exam I) was 5.0 ± 6.0 (Table 1).

**Table 1:** Descriptive statistics of study variables (mean ± SD) SD: standard deviation. Score gain=Exam II − Exam I.

Variable	Mean ± SD
Questions generated	31.5 ± 20.2
Sessions	13.6 ± 5.7
Total usage time (min)	153.1 ± 112.9
Exam I score	17.1 ± 4.4
Exam II score	22.1 ± 7.6
Score gain (Exam II – Exam I)	5.0 ± 6.0

Pre-post comparison

Paired t-tests revealed significant examination performance improvements. The mean score for Exam II was significantly higher than that for Exam I (mean gain=5.0 ± 6.0, t-statistic=4.40, degrees of freedom=27, p<0.001), corresponding to a large effect size (Cohen’s d=0.83).

Correlation analysis

Pearson’s correlation analysis revealed weak-to-moderate positive associations between exam performance and learning log indicators. Exam II scores were significantly correlated with the number of questions generated (r=0.39, p<0.05) and exhibited a marginal association with the number of sessions (r=0.36, p=0.08). Score gains (Exam II − Exam I) were positively but not significantly associated with the number of questions generated (r=0.31, p=0.11) and the number of sessions (r=0.36, p=0.08). No significant associations were observed between the total usage time and exam performance (Table 2).

**Table 2:** Pearson’s correlation coefficients between exam performance and learning log indicators Values are Pearson’s correlation coefficients (r). † p < 0.10; * p < 0.05; ** p < 0.01. Score gain=Exam II − Exam I.

Indicators	Score Gain	Questions Generated	Sessions	Total Usage Time	Exam I	Exam II
Score gain	1.00	0.31	0.36 †	0.18	0.03	0.81 **
Questions generated		1.00	0.66 **	0.39 *	0.22	0.39 *
Sessions			1.00	0.44 *	0.14	0.36 †
Total usage time				1.00	0.18	0.28
Exam I					1.00	0.61 **
Exam II						1.00

Cluster analysis

Ward’s hierarchical cluster analysis identified three distinct learning behavior patterns. Cluster 1 (n=14) was characterized by low engagement, with below-average standardized scores across all indicators (questions, sessions, and time). Cluster 2 (n=9) demonstrated moderate engagement, generating more questions (39.7 ± 25.8) and sessions (16.4 ± 8.4) than Cluster 1, while spending an average amount of time (161.7 ± 34.6 min). Cluster 3 (n=6) generated slightly fewer questions (34.8 ± 24.3) and sessions (15.5 ± 5.4) than Cluster 2, while spending substantially more time (328.3 ± 62.1 min).

**Figure 1:**
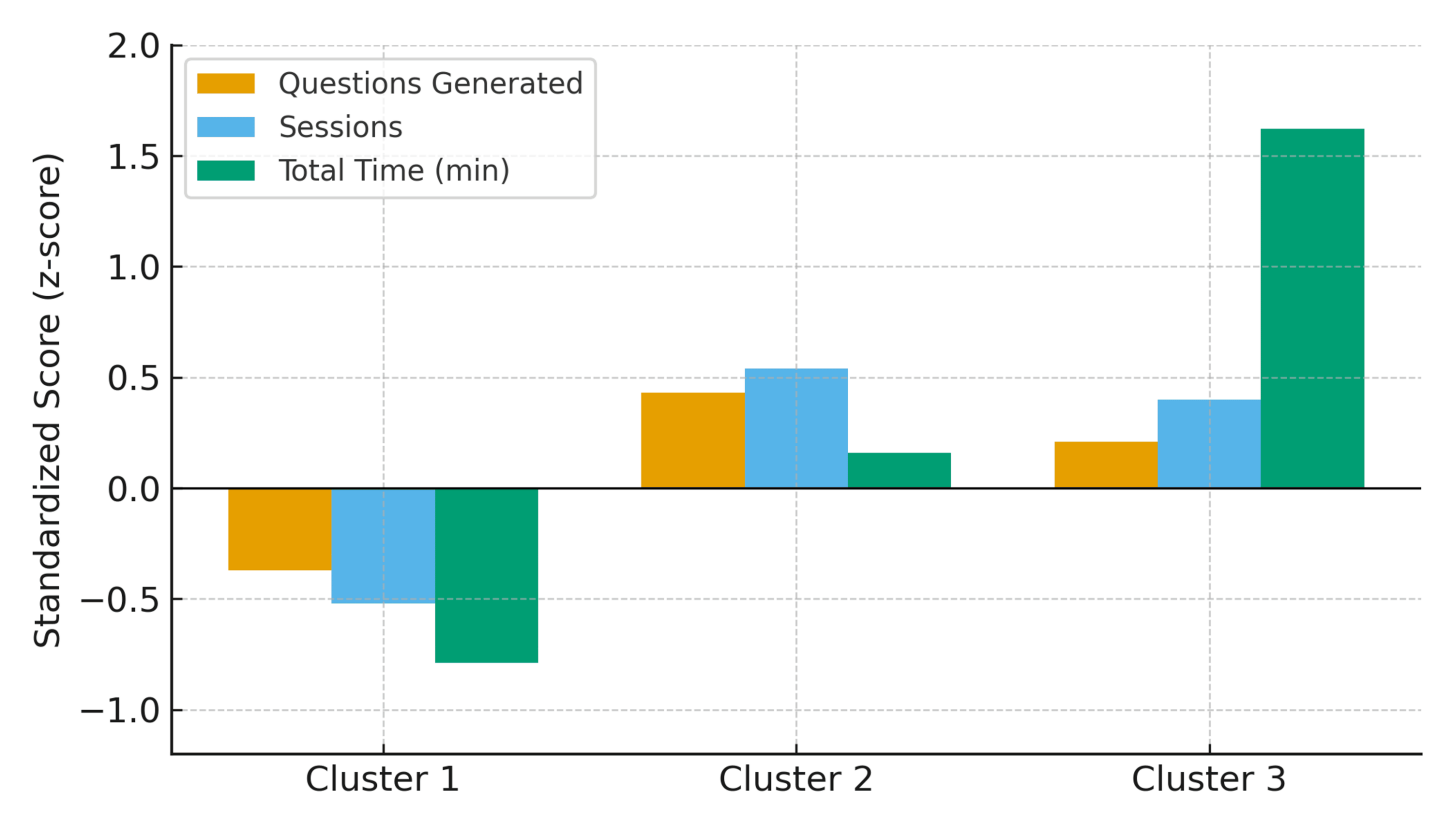
Standardized scores of learning log indicators by cluster Negative values indicate below-average performance relative to the overall sample.

Descriptive statistics of exam performance and learning log indicators for each cluster are presented in Table 3. Cluster 1 exhibited a mean score gain of +5.5 ± 3.2, Cluster 2 exhibited +1.8 ± 7.4, and Cluster 3 exhibited +8.8 ± 6.8. The standardized scores for the three learning indicators are summarized in Table 4, and their relative differences across the clusters are illustrated in Figure 1.

**Table 3:** Descriptive statistics of exam performance and learning log indicators by cluster SD: standard deviation. Gain=Exam II − Exam I.

Variable	Cluster 1 (n=14)	Cluster 2 (n=9)	Cluster 3 (n=6)
Exam I (mean ± SD)	15.5 ± 2.8	19.2 ± 6.1	17.5 ± 3.6
Exam II (mean ± SD)	20.6 ± 5.0	21.0 ± 10.6	26.3 ± 6.7
Gain (Exam II – I)	5.5 ± 3.2	1.8 ± 7.4	8.8 ± 6.8
Questions generated	22.5 ± 15.1	39.7 ± 25.8	34.8 ± 24.3
Number of sessions	9.5 ± 3.7	16.4 ± 8.4	15.5 ± 5.4
Total time (min)	53.4 ± 33.2	161.7 ± 34.6	328.3 ± 62.1

**Table 4:** Standardized scores (Z-scores) of learning log indicators by cluster Values are standardized z-scores. Negative values indicate below-average performance relative to the overall sample.

Variable	Cluster 1	Cluster 2	Cluster 3
Questions generated	–0.37	+0.43	+0.21
Number of sessions	–0.52	+0.54	+0.40
Total time	–0.79	+0.16	+1.62

## Discussion

This study explored OT students’ learning behaviors over one month using a custom-built ChatGPT-based question-generating bot. Three main findings were observed: First, mock examination scores improved after the intervention, with a large effect size. Second, correlations between usage logs and performance were weak to moderate, with the number of questions generated and the number of sessions exhibiting some association with exam outcomes; however, total usage time alone did not demonstrate a clear relationship. Third, cluster analysis identified distinct patterns of engagement, with the group that invested the greatest total usage time achieving the largest score improvements, despite generating only moderate numbers of questions and sessions.

These findings suggested that the quality of engagement, rather than the quantity of questions generated, plays an important role in exam preparation. Although some associations were observed between question counts and sessions, the cluster that generated the most questions exhibited only a modest improvement. Conversely, students who spent substantially more time with the bot achieved the greatest gains. This suggests that how students engaged, such as carefully reviewing explanations and verifying uncertain outputs, may have been more influential than the volume of practice alone. Such behaviors align with the importance of self-directed and reflective learning strategies emphasized in health profession education [12-14].

Another interpretation concerns the role of hallucinations in AI output; LLMs occasionally generate inaccurate or misleading information [15,16]. Participants in the high-time cluster may have answered the generated questions while also evaluating the accuracy of the outputs and seeking confirmation from textbooks or other reliable sources when inconsistencies arose. This process of questioning, verifying, and consolidating knowledge can partially explain the larger performance gains. Although hallucinations are generally regarded as a limitation of AI, they may also serve as a stimulus for deeper and more critical engagement with learning materials in educational contexts.

The educational implications of these findings are noteworthy. AI-based tools can support diverse learning strategies and allow students to individualize their study approaches. Previous studies in OT and related fields reported educational utility such as enhancing creativity, realism, and student engagement [5,10,11]. This study contributes to these studies by illustrating distinct engagement patterns and showing that not all usage behaviors contribute equally to learning outcomes.

This study had several limitations. First, this study had a one-group pre-post design without a control group, and improvements cannot be attributed solely to ChatGPT use. Second, the intervention period was limited to one month, and the sample size was modest, involving a single class cohort. Third, the learning logs were self-reported, which may have introduced recall or reporting bias. Fourth, the sample size was small, and the clustering was exploratory, guided primarily by interpretability and visual inspection of the dendrogram. Therefore, statistical comparisons between clusters were not conducted, and the findings should be interpreted with caution.

Future studies should employ predefined analytical models with quantitative criteria (e.g., silhouette coefficient) to confirm the reproducibility of these findings. Moreover, larger samples and more rigorous designs, including controlled or crossover trials, are needed to examine how different usage patterns influence learning outcomes. In particular, the students in Cluster 3, who invested the greatest total usage time and achieved the largest gains, warrant closer examination. Qualitative studies may provide insights into how these students engaged with the bot, how they addressed uncertain or inaccurate outputs, and which strategies may have contributed to their performance improvement. These findings can inform educational models that guide broader student populations.

## Conclusions

This exploratory study examined how OT students used a custom-built ChatGPT-based question-generating bot to prepare for the national licensure exam. Examination scores improved after one month of use, and cluster analysis identified distinct learning patterns, with qualitative aspects of engagement appearing more closely related to performance gains than practice volume. These findings emphasized the importance of guiding students not only in accessing AI-generated content but also in critically engaging with it. Future studies with controlled designs and qualitative methods are warranted to clarify effective strategies for integrating AI into health profession education. Notably, this study’s insights may inform broader approaches for incorporating AI tools into clinical education.

## Figures and Tables

**Figure 2:**
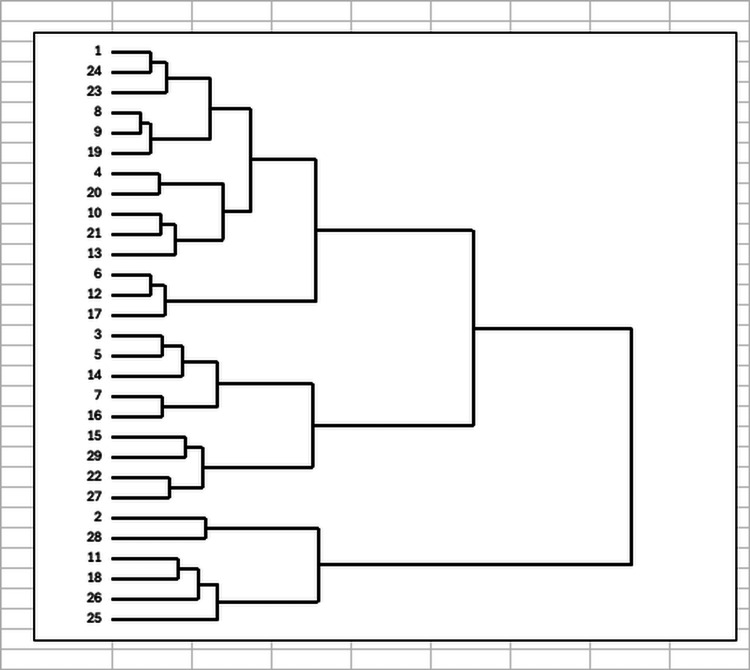
Dendrogram of learning behavior clusters The dendrogram was generated using Ward’s hierarchical clustering method based on standardized log variables (number of questions, number of sessions, total time). It was used to determine the number of clusters by inspecting the tree structure, interpretability, and sample size considerations. The figure included in the appendix was created by the authors using HAD statistical software (version 17.0; Shimizu, H., Kwansei Gakuin University, Japan) based on the original data obtained in this study.

## Appendices

Appendix 1

User guide for the ChatGPT-based question-generating bot

1) Purpose of this activity

Strengthen the three basic subjects (anatomy, kinesiology, and physiology) over one month and aim for improved performance in the second mock examination.

Students are expected to generate at least 15 questions per week, with additional practice encouraged, if possible.

2) Getting Started - Three Steps (approx. 5 minutes)

Download the ChatGPT application (iOS/Android recommended; web browser also acceptable).

Create a free account (email + SMS authentication; Plus subscription is not required).

Access the bot via the provided URL and press “Open GPT” to begin.

Bot URL: https://chatgpt.com/g/g-WUUqLkOVY-zuo-ye-liao-fa-shi-guo-jia-shi-yan-wen-ti-lian-xi-yong

3) How to Use the Bot and Rules

Enter up to three keywords (e.g., “ADL, stroke, evaluation”) to generate a five-option MCQ with an explanation.

AI outputs are not always accurate. Students must cross-check textbooks and guidelines.

The weekly target was at least 15 generated items (one question + answer counts as one item).

Do not delete conversation logs; they must be preserved for subsequent submission.

Do not enter or generate personally identifiable or patient-specific information.

4) Review and Use of Logs

When logged in, past interactions remain in “History.” Students must review incorrect items.

It is recommended to note corrections or additional references to improve learning efficiency.

5) Weekly Report Submission (via Google Forms)

Deadline: every Sunday at 23:59.

Report items: number of generated questions, total study time, AI accuracy, and extent of independent verification

The Google Form URL will be posted in Google Classroom.

6) Support

Please contact the course instructors for questions or technical issues.

Key Points Summary

Install the application and access the bot via the link.

Aim to generate at least 15 questions per week.

Always verify AI answers using textbooks or guidelines.

Keep chat logs and submit them later as instructed.

Contact instructors if any issues arise.

Appendix 2

Dendrogram of Learning Behavior Clusters
